# Case report: The promising application of dynamic functional connectivity analysis on an individual with failed back surgery syndrome

**DOI:** 10.3389/fnins.2022.987223

**Published:** 2022-09-23

**Authors:** Jingya Miao, Isaiah Ailes, Laura Krisa, Kristen Fleming, Devon Middleton, Kiran Talekar, Peter Natale, Feroze B. Mohamed, Kevin Hines, Caio M. Matias, Mahdi Alizadeh

**Affiliations:** ^1^Department of Neurosurgery, Thomas Jefferson University, Philadelphia, PA, United States; ^2^College of Osteopathic Medicine of the Pacific, Western University of Health Sciences, Pomona, CA, United States; ^3^Sidney Kimmel Medical College, Philadelphia, PA, United States; ^4^Department of Neurological Surgery, Thomas Jefferson University, Philadelphia, PA, United States; ^5^Department of Occupational Therapy, Thomas Jefferson University, Philadelphia, PA, United States; ^6^Department of Radiology, Thomas Jefferson University, Philadelphia, PA, United States

**Keywords:** failed back surgery syndrome, dynamic functional connectivity, resting state fMRI, spinal cord stimulation, case report

## Abstract

Failed back surgery syndrome (FBSS), a chronic neuropathic pain condition, is a common indication for spinal cord stimulation (SCS). However, the mechanisms of SCS, especially its effects on supraspinal/brain functional connectivity, are still not fully understood. Resting state functional magnetic resonance imaging (rsfMRI) studies have shown characteristics in patients with chronic low back pain (cLBP). In this case study, we performed rsfMRI scanning (3.0 T) on an FBSS patient, who presented with chronic low back and leg pain following her previous lumbar microdiscectomy and had undergone permanent SCS. Appropriate MRI safety measures were undertaken to scan this subject. Seed-based functional connectivity (FC) was performed on the rsfMRI data acquired from the FBSS subject, and then compared to a group of 17 healthy controls. Seeds were identified by an atlas of resting state networks (RSNs), which is composed of 32 regions grouped into 8 networks. Sliding-window method and k-means clustering were used in dynamic FC analysis, which resulted in 4 brain states for each group. Our results demonstrated the safety and feasibility of 3T MRI scanning in a patient with implanted SCS system. Compared to the brain states of healthy controls, the FBSS subject presented very different FC patterns in less frequent brain states. The mean dwell time of brain states showed distinct distributions: the FBSS subject seemed to prefer a single state over the others. Although future studies with large sample sizes are needed to make statistical conclusions, our findings demonstrated the promising application of dynamic FC to provide more granularity with FC changes associated with different brain states in chronic pain.

## Introduction

Failed back surgery syndrome (FBSS) is a chronic neuropathic condition characterized by chronic leg or lower back pain (cLBP) following one or more lumbar spinal surgeries (Thomson, 2013). Literature has reported the incidence of FBSS in up to 40% of the cases (Alizadeh and Sharifzadeh, 2021). Disabling neuropathic pain usually persists or worsens with time and dramatically affects quality of life (Mondello et al., 2018). When other therapies have failed, treatment paradigms resort to spinal cord stimulation (SCS), which provides electric therapy through implanted electrodes for pain relief (Alizadeh and Sharifzadeh, 2021; Hosseini et al., 2021). In standard clinical practice, a subjective trial phase is carried out to determine patients who will benefit from SCS therapy before the permanent implantation of the device (North et al., 2020; De Andres et al., 2021). In the literature, successful trials varies from 40 to 80%, which is influenced by patient’s age, SCS program settings, pain localization and patient’s history of spine surgery (Odonkor et al., 2020). In addition, a third of patients who had failed SCS trial may still benefit from permanent implant in a 6–18 months follow-up (Oakley et al., 2008), yet patients with successful trial may show decreased efficacy in a 4 years follow-up (Teton et al., 2019). Therefore, despite the clinical efficacy of SCS, its mechanisms of pain relief and patient selections are still not completely understood. Both spinal segmental and supraspinal pathways are complementary systems in the effectiveness of SCS (El-Khoury et al., 2002; Barchini et al., 2012; De Groote et al., 2020b).

Functional magnetic resonance imaging (fMRI) studies in patients suffering from cLBP have shown alterations of brain activity related to pain perception and emotion (Hashmi et al., 2013; Sun et al., 2021). Resting state fMRI (rsfMRI) is a powerful tool for mapping the intrinsic brain functional connectivity (FC). Previous studies have observed widespread changes in cLBP patients, highlighting the disrupted default mode network (DMN) along with elevated FC related to the medial prefrontal cortex, anterior insula, amygdala, and sensorimotor regions (Kregel et al., 2015; Jiang et al., 2016; Mao et al., 2022a,b). Studies that focused on the FBSS patient population demonstrated generally decreased connectivity within the DMN (Kornelsen et al., 2013) and increased connectivity associated with multiple resting state networks (RSNs), including the salience, central executive, and sensorimotor networks (Kolesar et al., 2017). Although phantom studies have demonstrated safety of SCS stimulation *via* externalized neurostimulators in both 1.5 and 3T MRI (Moens et al., 2012), only a few studies have looked into the effects of SCS on resting state functional connectivity in FBSS. To our knowledge, only two research groups (Moens and Rezai) have published studies on rsfMRI of FBSS patients undergone permanent SCS implantation (Deogaonkar et al., 2016; De Groote et al., 2019, 2020a). SCS systems have received FDA approval for 1.5T MRI; however, it remains off-label to scan under 3T MRI (Thornton, 2017). With careful considerations of all the parameters of FDA guidelines (SAR, B0 values, head-only transmit, and scan duration), we conducted 3T rsfMRI in a single FBSS patient, who had received permanent SCS implantation. In this case study, we demonstrate the safety of 3T MRI in a patient with a permanently implanted SCS to provide evidence for future studies.

Increasing evidence suggests that spontaneous brain network communication measured *via* rsfMRI is intrinsically dynamic. FC among resting state networks (RSNs) have been demonstrated to have dynamic fluctuations rather than stationary throughout the entire scan time, especially relevant to the cognitive and attentional functions (Allen et al., 2014). A sliding-window technique followed by k-means clustering has been applied for the assessment of the dynamic characteristics; the resulting dynamic states (or brain states) represent highly replicable sub-patterns of FC during resting state (Allen et al., 2011, 2014). With such progress in neuroimaging analysis, Kucyi and Davis first introduced the concept of dynamic pain connectome in the brain (Kucyi and Davis, 2015). In patients suffering from low-back-related leg pain, temporal variabilities were found increased from healthy control groups (Pei et al., 2020). Additionally, immediate changes of rsFC were observed in cLBP after a pain-inducing maneuver and a manual therapy (Yu et al., 2014; Isenburg et al., 2021). Therefore, the second aim of this case study was to evaluate our hypothesis that FBSS presents alterations in dynamic FC from healthy controls. Such findings may be helpful in identifying potential biomarkers in the FBSS patient population and/or the supraspinal effects of SCS.

## Case description

A 46-year-old female, who had right L5-S1 microdiscectomy and revision microdiscectomy 4 and 5 months earlier, presented with chronic low back and leg pain. Her pain was described as constant burning sensation radiating unilaterally down the right lower extremity, with accompanying numbness and weakness in her right leg. Her pain would get worse as the day extended. At the time of the initial presentation, an 8/10 visual analog scale (VAS) was rated, and the Oswestry Disability Index (ODI) was scored 26/50 (modified score: 23). As no significant nerve root compression was noted on the patient’s lumbar MRI, she was diagnosed with FBSS. After her initial evaluation and non-operative therapy (including aquatherapy, physical therapy, chiropractor care, acupuncture, and epidural steroid injection), it was recommended that she consider SCS for her chronic pain reduction. All risks and benefits of each therapy (both non-operative and SCS) were explained to the patient. She had a successful SCS trial (8 days) and experienced approximately 70% pain relief with improvements in activity tolerance. The patient felt most pain relief while on differential target multiplexed (DTM) programming. As such, permanent SCS implantation was performed 2 weeks later. Following standard clinical care, two SCS permanent percutaneous electrodes (Medtronic, Inc., Minneapolis, MN, USA) were implanted in patient’s thoracic epidural space. One electrode was positioned at the top of T8 and the other electrode in the mid body of T9, so that they were at the anatomical midline with contacts of leads covering T8-10. A left buttock incision was made for the placement of a rechargeable implantable pulse generator (Medtronic Intellis Neurostimulator). The surgery was successful with no complications.

During her follow-up visit 2 weeks after her permanent SCS implantation and before the start of SCS treatment, the patient noted well-controlled incisional pain without the need of any additional pain medication. She reported an 8/10 VAS and was on her regular pain medications (Duloxetine, 60 mg by mouth daily; Gabapentin, 600 mg by mouth 2 times a day and 1,200 mg at bedtime; Percocet, 5–325 mg tablet, 1 tablet by mouth every 4 h as needed for up to 3 days; Naloxone, 4 mg per actuation spray, 1 spray as needed). Other clinical assessments were also collected, including the ODI, Pain Catastrophizing Scale, Hospital Anxiety Scale, Hospital Depression Scale, Pittsburgh Sleep Quality Assessment, and Central Sensitization Inventory (Table 1). rsfMRI was acquired on the same day. SAR values was monitored throughout the scan, which stayed way below the Medtronic MR guidelines and FDA regulations (3 W/kg head only). The patient was closely monitored and did not report any discomfort during and after the MRI acquisition process. She was also checked by a neurosurgeon with no complications or side effects noticed due to MRI. These measurements will serve as the baseline for future follow-up visits for monitoring the SCS therapy.

**TABLE 1:** Clinical assessments of the failed back surgery syndrome (FBSS) patient collected after permanent spinal cord stimulation (SCS) implantation and before the onset of SCS therapy.

Assessments	Overall score	Average score (score scale)	Percentage	Significance
Oswestry disability index (ODI)	30/50	3 (0–5)	60%	Severe disability
Pain catastrophizing scale	39/52	3 (0–4)	75%	Experiencing pain to a great degree
Hospital anxiety scale	9/21	1.3 (0–3)	43%	Borderline abnormal
Hospital depression scale	13/21	1.9 (0–3)	62%	Abnormal
Pittsburgh sleep quality assessment	9	1.8 (0–3)	–	Indicate poor sleep quality
Central sensitization inventory	37/100	1.5 (0–4)	37%	Symptoms are unlikely due to central sensitivity syndromes

### Ethical statement

This prospective study was approved by the local Institutional Review Board. Written informed consent was obtained from the FBSS participant, as well as all healthy volunteers.

Although 3T MRI is still off label, safety controls were carefully addressed prior to conducting the study. In addition to phantom tests (Moens et al., 2012), previous clinical studies have shown safety of 3T MRI on their SCS patient cohorts without any complications, using GE MR 750w Discovery 3T (De Groote et al., 2019) and Philips Achieva 3T MRI scanner (Deogaonkar et al., 2016; De Groote et al., 2020a). The later scanner was the same device model that we would use in this study. Moreover, complying with the FDA guidance, the specific absorption rate (SAR) and the B1rms values were below the recommended values to avoid lead heating. In addition, transmit-receive head coil was used to lower risk of RF-transmit related heating due to the implant located in the lower thoracic region. The patient was screened for safety before entering the MR scan room, and the stimulation was kept OFF throughout the scanning duration, which was limited to less than 30 min. The patient was completely aware of all potential risks and decided to participate. She didn’t report any discomfort or adverse effects due to the imaging.

## Diagnostic assessment

### Magnetic resonance imaging acquisition

During the 2-week follow-up visit, rsfMRI was scanned with stimulation-OFF on a 3.0T Philips Achieva MR scanner. The scanner settings for rsfMRI were: FOV = 24 cm, TR = 2,000 ms, TE = 25 ms, flip angle 90-degree, voxel size 3 × 3 × 3 mm, axial slices = 40, matrix size = 96 × 96. The patient was instructed to keep eyes closed and relaxed without falling asleep during the 12 min (360 volumes) resting state scan. In addition, 17 healthy volunteers were recruited and underwent 6-min rsfMRI (180 volumes) with the same instructions and scanner settings.

### Functional connectivity analyses

All rsfMRI data was preprocessed using the default pipeline provided in CONN toolbox version 20b (RRID:SCR_009550).^1^ For each resting state session, the first 10 volumes were removed, followed by functional images realignment, unwarping, slice-timing correction, outlier identification, segmentations (including gray matter, white matter, CSF, and CONN’s default resting state networks), and normalization to the MNI standard space (resolution 2 mm × 2 mm × 2 mm), and smoothing with a Gaussian kernel of 6 mm full width half maximum (FWHM). Functional images were then denoised using the component-based noise correction method provided in CONN (Behzadi et al., 2007). The temporal time series of estimated head motion in 6 degrees of freedom and the BOLD signals within the subject-specific white matter and CSF segments were regressed out. The resulting BOLD time series were filtered using a band pass of 0.01–0.15 Hz.

The cleaned BOLD signal time series were used for the following assessments *via* seed-based static and dynamic FC. The regions of interest (ROIs) were identified using CONN’s default network atlas, which includes 32 cortical brain regions grouped into eight networks. Static FC (sFC) were calculated by taking into account the entire scanning period using Pearson’s correlation between each pair of the 32 ROIs. A sFC matrix was obtained for each subject. Then, the 17 healthy controls’ sFC matrices were averaged and compared to the sFC matrix obtained from the FBSS patient.

**FIGURE 1:**
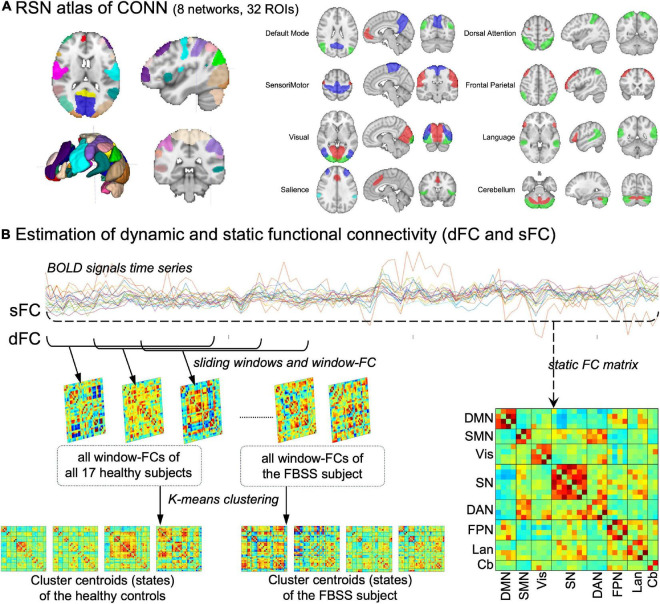
Method pipeline. **(A)** The resting state network (RSN) atlas in CONN toolbox, which composed of 32 ROIs grouped into 8 networks. **(B)** Pipeline of the estimations of dynamic and static functional connectivity (dFC, sFC). DMN, default mode network; SMN, sensorimotor network; Vis, visual network; SN, salience network; DAN, dorsal attention network; FPN, frontal parietal network; Lan, language network; Cb, cerebellum.

Dynamic FC (dFC) was assessed using a sliding window method, followed by k-means clustering (Allen et al., 2011, 2014). A short time length, called a window (window size = 30 TRs = 60 s), was used to calculate corresponding FC matrix within that period. This window then slides down the timeline in 1 TR (=2 s) step to get a second window and its corresponding FC matrix. This was repeated until the last window covering the last 30 TRs of each session. This resulted 140 windows for each healthy volunteer and 320 windows for the FBSS subject. The elbow criterion (defined as the ratio of intra-cluster distance to inter-cluster distance) was used to estimate the optimal number of clusters (K) and k-means clustering was applied to group window-matrices into K dynamic states. Clustering was performed separately for the healthy control group and the FBSS subject, given the large difference of numbers of windows between these two groups. Every state matrix represents the centroid of a cluster, and quantitative metrics of dFC were calculated for each group. The fraction rate refers to the occurrence frequency of each dynamic state; and the mean dwell time is defined as the consecutive number of windows in each dynamic state. All of the dFC procedures were undertaken using MATLAB-based GIFT toolbox (Allen et al., 2011, 2014). The analysis pipeline is shown in Figure 1.

### Results of static and dynamic functional connectivity

**FIGURE 2:**
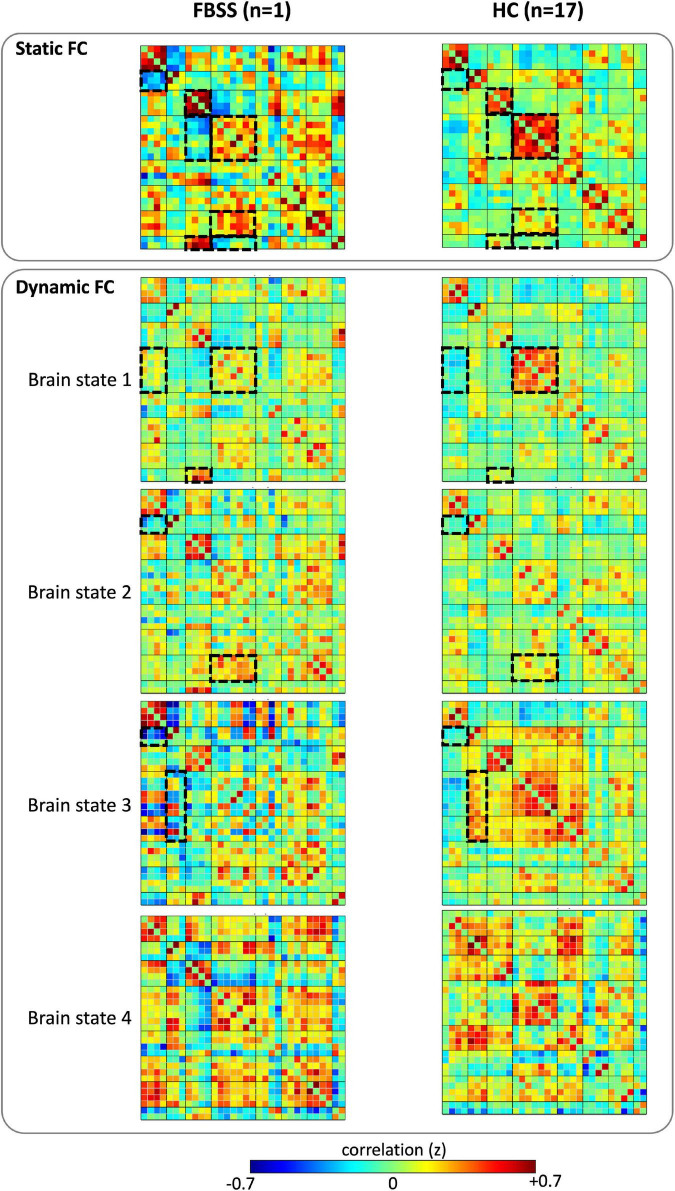
Static functional connectivity (FC) matrices and the cluster centroids of the dynamic FC matrices of the FBSS subject and the healthy controls (HC). Dashed boxes highlighted the distinct differences of the FC patterns observed between the FBSS patient and the HC groups.

Compared to the sFC matrix of the healthy controls (HCs), our subject’s sFC presented with widespread altered FCs both within and between RSNs. In summary, the differences between our subject and HCs are: (1) decreased FC within salience network; (2) decreased FC connectivity between DMN and sensorimotor, salience and visual, salience and cerebellum; (3) increased FC within the visual network; and (4) increased FC between visual and cerebellum, as well as salience and language (Figure 2).

**FIGURE 3:**
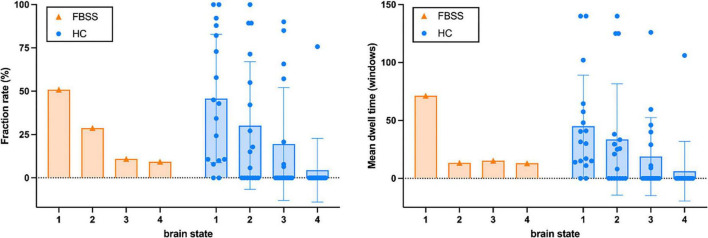
The fraction rate (%) and mean dwell time (number of windows) of each brain state calculated from the FBSS subject and the healthy controls (HC).

The dFC results showed that all four dynamic state patterns of the FBSS subject were distinguishable from those of the healthy controls. For brain state 1 and 2, the FBSS subject showed relatively similar patterns to the healthy controls, whereas for brain state 3 and 4, the FBSS subject showed distinct patterns. Increased FC of the salience network coupling with the DMN, language, and visual networks were observed in the FBSS subject. Decreased FC of the sensorimotor network coupling with the DMN, salience, dorsal attention networks were seen in the FBSS subject (Figure 2). Quantitative metrics of dFCs revealed that FBSS patient seemed to have a trending of increased mean dwell time in a single dynamic state, whereas the healthy group showed relatively more distributed mean dwell time across the dynamic states (Figure 3).

## Discussions

In this case study, we scanned a FBSS patient with SCS system implanted (off-stimulation) on a 3T MRI device with careful safety considerations. Being part of an ongoing prospective study, our first case demonstrated the safety and feasibility of 3T MRI scanning for patients with implanted SCS system. The rsfMRI data in this study will also serve as the baseline for intra-subjective studies about the effects of SCS therapy on supraspinal FC in FBSS. Here, we assessed the resting state network connectivity using both static and dynamic FC analyses and compared to healthy controls. The static FC pattern from the FBSS patient showed wide-spread alterations among all the RSNs. The dynamic FC analysis clustered all window-matrices, separately for each group, into four brain states. Visual evaluation of the matrix patterns revealed that that brain state 1 and 2 of both groups had relatively similar patterns compared to brain state 3 and 4. Similar fraction rates were observed in both groups: about half of window-matrices occurred as brain state 1. Regarding the mean dwell time shown by the bar graph, the FBSS case seemed to prefer a single state (State 1) over the other three states. As there’s only one case in the FBSS group, future statistical analysis with larger sample size is necessary to make any concrete conclusions.

Increasing evidence suggest the temporal fluctuations of brain FC in chronic pain and warrant the necessity to consider the dynamic characteristics of pain connectome in contemporary research (Kucyi and Davis, 2015; Pei et al., 2020). Effective treatments of cLBP have shown the ability to reverse abnormal FCs toward healthy controls (Seminowicz et al., 2011; Li et al., 2014), indicating the plasticity of the pain connectomes. Changes of rsFC were also observed in cLBP subjects after 10 min pain-inducing maneuver, which further demonstrated the dynamic characteristics of FC in chronic pain (Yu et al., 2014). In a previous dFC study of a group of healthy participants, it was demonstrated that pain perception threshold was associated with the frequency of a DMN-dominant brain state, whereas the touch threshold was associated with another sFC-like brain state (Yuan et al., 2019). Other dFC studies found that the mean dwell time in a less frequent brain state, out of 5–6 total states, was significantly higher in the chronic migraine group versus healthy controls (Zou et al., 2021) and in subjects with post-brain-traumatic headache vs. those without (Li et al., 2021). Pei et al. applied the sliding-window method (without clustering) and revealed a higher temporal variation of window-matrices in subjects with low-back-related leg pain compared to healthy controls (Pei et al., 2020). The current case study demonstrated with qualitative results the promising application of dFC in FBSS, as well as cLBP, populations. Moreover, we observed different FC patterns between the two groups in the less frequent brain state 3 and 4, indicating the potential of dFC analysis to detect more sensitive and specific differences between FBSS and HCs.

A number of studies have used rs-fMRI to assess alterations of the intrinsic brain functional connectivity in cLBP compared to healthy controls. Widespread findings have been found in the current literature. In general, cLBP patients showed decreased FC within the DMN (Kornelsen et al., 2013; Jiang et al., 2016; Ayoub et al., 2019; Ng et al., 2021a), along with increased FC between the DMN and other networks, including the emotional, cognitive, and/or central executive networks (Kornelsen et al., 2013; Jiang et al., 2016; Ma et al., 2020; Ng et al., 2021b; Mao et al., 2022b,c) in static FC studies. Particularly, medial prefrontal cortex (mPFC) seems playing an important role in cLBP (Baliki et al., 2011, 2014; Yu et al., 2014). The lack of brain activities in common noxious regions indicated that chronic pain may not be caused by nociceptive process, but rather associated with emotional and cognitive processes and potentiated through connectivity of these regions (Ng et al., 2017; Pahapill et al., 2020). In addition, enhanced rsFC in cLBP were also observed in the sensorimotor networks (Mao et al., 2022a), visual networks (Shen et al., 2019; Lamichhane et al., 2021a,b), reward network (Yu et al., 2020), and DMN-PAG couplings (Yu et al., 2014). Given the widespread findings in rsFC patterns, machine-learning, although on small sample size, showed some capabilities to differentiate cLBP patients from healthy controls (Tu et al., 2019; Lamichhane et al., 2021a,b). These reports were based on heterogeneous cLBP populations with different etiologies (neuropathic vs. nociceptive).

For homogeneous cohorts, studies that focused on the FBSS population revealed similar alterations in cortical networks (i.e., decreased FC within the DMN, and increased FC between the DMN and salience, central executive, and sensorimotor networks) compared to healthy controls (Kornelsen et al., 2013; Kolesar et al., 2017; Pahapill et al., 2020). Moreover, Pahapill et al. reported decreased FC between the striatum and other networks, decreased FC between the PAG and DMN, and increased FC between the PAG and sensorimotor networks (Pahapill et al., 2020). There is shortage of rs-fMRI studies that specifically exams homogeneous groups, hence it remains unclear whether neuropathic chronic pain express differentiable connectivity patterns from nociceptive chronic pain patients. However, a recent EEG study measured the brain responses to nociceptive stimulation and revealed significant differences in coupling strengths between patients with nociceptive and neuropathic etiologies. Their findings provided positive evidence for finding potential neuroimaging biomarkers of cLBP subtypes (Goudman et al., 2020). We believe that dynamic FC has the potential to provide new biomarkers in future studies in FBSS, better patient selections for SCS treatments, and/or the mechanisms of SCS.

## Limitations

There are several limitations that should be considered in this case study. First, only a single case was analyzed and compared with healthy controls, that was not age-matched to the case. Although trends were observed between the FBSS case and healthy controls, statistical analysis were lacking due to the sample size. Second, the ROIs were extracted based on a pre-determined atlas including 8 RSNs in the cerebral area, without subcortical regions. Recent studies that explored the effects of SCS for FBSS patients have shown FC alterations in the descending pain modulatory system (DPMS), which includes the periaqueductal gray (PAG) and the rostral ventromedial medulla (RVM) in the brainstem (De Groote et al., 2019). Our study would likely not capture those effects given that active stimulation therapy had not occurred in the implant yet. Only changes related to the 8-day trial therapy may have been captured, if contributory. Nonetheless, this study demonstrated that the quality of MR images was comparable with those acquired from patients without SCS implants, which did not limit the study of subcortical and brainstem regions. The implanted electrode and pulse generator were located with sufficient distance from the brain to not introduce artifact in the imaging region. Therefore, we are planning to conduct a larger study in the future.

## Conclusion

This case study demonstrated that, with prior safety considerations, patients with SCS implantation (during off-stimulation) may safely undergo 3T MRI. The preliminary study of a FBSS patient provided support for our ongoing prospective study, which aims to explore the effects of SCS on brain functional connectivity. Our results also demonstrated that dynamic FC in fMRI studies in chronic pain provides more granularity with FC changes associated with different brain states. Further studies with larger sample sizes and inclusions of subcortical brain regions are warranted to better understand the brain functional connectomes in subtypes of chronic pain, as well as the mechanisms of SCS therapy. In addition, future studies should compare changes in FC over time in patient undergoing SCS therapy.

## Data availability statement

The datasets presented in this article are not readily available because institutional regulations require the establishment of formal data sharing agreements before patient identifying information included in this dataset or metadata can be shared. Deidentified raw data can be provided upon request to the corresponding author, after approval from the institutional review board. Requests to access the datasets should be directed to JM, jingya.miao@jefferson.edu.

## Ethics statement

The studies involving human participants were reviewed and approved by the Thomas Jefferson University Institutional Review Boards. The patients/participants provided their written informed consent to participate in this study.

## Author contributions

JM and MA contributed to the concept and design of the study. IA, LK, KF, DM, KT, and PN helped in the acquisition and verification of data. JM, IA, FM, KH, CM, and MA interpreted the data. JM drafted the manuscript and has full access to all the data in the study and responsible for the integrity of the data and the accuracy of the data analysis. FM, KH, CM, and MA critically revised the manuscript for important intellectual content. MA supervised the study. All authors contributed to the article and approved the submitted version.

## Footnotes

1
www.nitrc.org/projects/conn
